# Reducing medical device alarms by an order of magnitude: A human factors approach

**DOI:** 10.1177/0310057X20968840

**Published:** 2021-02-02

**Authors:** Erik Koomen, Craig S Webster, David Konrad, Johannes G van der Hoeven, Thomas Best, Jozef Kesecioglu, Diederik AMPJ Gommers, Willem B de Vries, Teus H Kappen

**Affiliations:** 1 Department of Paediatrics, Paediatric Intensive Care, Wilhelmina Children’s Hospital, Academic Medical Centre Utrecht, Utrecht, The Netherlands; 2 Department of Anaesthesiology and Centre for Medical and Health Sciences Education, University of Auckland, Auckland, New Zealand; 3 Department of Perioperative Medicine and Intensive Care at Karolinska University Hospital, Stockholm, Sweden; 4 Department of Intensive Care Medicine, Radboud Academic Medical Centre, Nijmegen, The Netherlands; 5 Department of Critical Care, King’s College Hospital, London, UK; 6 Department of Intensive Care Medicine, Academic Medical Centre Utrecht, Utrecht, the Netherlands; 7 Department of Intensive Care Medicine, Erasmus Medical Centre, Rotterdam, The Netherlands; 8 Department of Neonatology, Academic Medical Centre Utrecht, Utrecht, The Netherlands; 9 Department of Anaesthesia, Intensive Care and Emergency, Academic Medical Centre Utrecht, Utrecht, The Netherlands

**Keywords:** ICU, alarms, alarm integration, future ICU design, comprehensive alarms, redesign device chain, patient safety, modern hospital, human factors, aviation.

## Abstract

The intensive care unit (ICU) is one of the most technically advanced environments in healthcare, using a multitude of medical devices for drug administration, mechanical ventilation and patient monitoring. However, these technologies currently come with disadvantages, namely noise pollution, information overload and alarm fatigue—all caused by too many alarms. Individual medical devices currently generate alarms independently, without any coordination or prioritisation with other devices, leading to a cacophony where important alarms can be lost amongst trivial ones, occasionally with serious or even fatal consequences for patients. We have called this approach to the design of medical devices the single-device paradigm, and believe it is obsolete in modern hospitals where patients are typically connected to several devices simultaneously. Alarm rates of one alarm every four minutes for only the physiological monitors (as recorded in the ICUs of two hospitals contributing to this paper) degrades the quality of the patient’s healing environment and threatens patient safety by constantly distracting healthcare professionals. We outline a new approach to medical device design involving the application of human factors principles which have been successful in eliminating alarm fatigue in commercial aviation. Our approach comprises the networked-device paradigm, comprehensive alarms and humaniform information displays. Instead of each medical device alarming separately at the patient’s bedside, our proposed approach will integrate, prioritise and optimise alarms across all devices attached to each patient, display information more intuitively and hence increase alarm quality while reducing the number of alarms by an order of magnitude below current levels.

## Introduction

Clinical alarms, patient monitoring systems and other medical devices are currently based on a single-device paradigm, in the sense that each device is designed to operate in isolation and to generate alarms independently at the patient’s bedside. In the context of modern high technology healthcare, where patients are typically connected to several devices, the single-device paradigm is outdated and routinely results in information overload, distraction for healthcare professionals and unnecessary audiovisual disturbances for patients. At the Academic Medical Centre Utrecht and Erasmus Medical Centre, the authors are working with equipment manufacturers in order to develop a practical solution to this problem which will substantially reduce the total number of alarms and integrate trivial alarms into comprehensive, more informationally meaningful ones—and this represents a paradigm shift in the way alarms in healthcare are currently organised. Taking the human factors principles which have been successful in eliminating alarm fatigue in commercial aviation, our approach involves applying these principles to the problem of alarm fatigue in healthcare—yielding the networked-device paradigm, comprehensive alarms and humaniform display of patient information. The networked-device paradigm involves connecting all medical devices in a virtual network for each patient which, in combination with a comprehensive alarm approach, can then be used to manage and prioritise all alarm signals issuing from individual devices. This approach is very different from the current single-device paradigm, where no management or prioritisation of alarms is possible between all the devices connected to each patient. Achieving such a global solution involves overcoming a number of technical hurdles, and we outline the approach we are currently pursuing in collaboration with equipment manufacturers. We believe this approach to the design of clinical alarms and medical devices will yield a system capable of safely catering for the healthcare needs of patients in modern hospitals. Our aim is first to implement such an approach in the intensive care unit (ICU), but the networked-device paradigm and comprehensive alarms are also equally applicable in operating theatres, hospital wards and any other care environment where medical devices are used.

## The problem of too many alarms in the intensive care unit

Audiovisual disturbances due to alarms in the ICU are well known, adversely affect the psychological wellbeing of patients, and significantly increase the workload for healthcare providers.^1–3^ The signalling mode of alarms is designed to attract the immediate attention of care providers by interrupting their current activities, resulting in cognitive stress.^4,5^ However, studies demonstrate that 90% of alarms require no action from healthcare providers.^6^ Noise levels in hospital ICUs have been recorded at a mean of 71.9 dBA, being equivalent to a busy office environment or the use of a vacuum cleaner in the room,^7^ with peak noise levels of 96 dBA,^8^ which is the equivalent of a propeller plane flyover at 150 metres. In addition, Darbyshire et al. reported that a significant proportion of loud sounds originate from equipment near patients’ ears.^4^

Technological advancements have transformed patient care in ICUs over recent decades. These advancements include complex physiological monitoring, continuous intravenous infusions of medications and invasive treatments such as mechanical ventilation, renal replacement therapy and extracorporeal life support. All these sophisticated technologies require continuous human oversight to monitor a patient’s condition, interpret the displayed information and adjust treatment accordingly. Yet, alarm design has changed little in this time, and safety and useability testing are conducted only at the level of the individual device, rather than considering safety and useability at the system level, comprising the many devices which are typically connected to each patient.^9^

Manufacturers of these technologies currently meet their legal and regulatory requirements by displaying as much of the information considered useful to healthcare providers as possible on each device, and creating the option to alarm every single parameter.^10–12^ Unfortunately, such sophisticated devices typically operate independently from each other. Without any coordination or prioritisation between devices, they collectively issue a stream of alerts and alarms that results in a cacophony where important alarms can be lost amongst trivial ones, as many alarms are caused by artifacts or are non-actionable. This quickly leads to alarm fatigue and the habit of switching off alarms in an attempt to manage them.^13,14^ Sampling the alarms from only the physiological monitors in the adult ICUs at the University Medical Centre Utrecht and Erasmus Medical Centre resulted in the recording of between 100 and 120 alarms per nurse per eight-hour shift—or an average of one alarm every four minutes. Adding the additional alarms of the ventilator, infusion pumps, renal replacement therapy or extracorporeal life-support devices would substantially increase this total. A recent study of this problem reported from a single hospital in the USA, with 77 intensive care beds, recorded the occurrence of an astonishing 2,558,760 unique physiological alarms during intensive care in a single month.^15^ Both the Joint Commission and the Emergency Care Research Institute (ECRI) have repeatedly identified alarm safety issues, including alarm fatigue, as patient safety hazards, which are known to be regularly associated with patient deaths. Between 2005 and 2010, 566 alarm-related patient deaths were reported to the US Food and Drug Administration.^16–18^

### Human factors design and the aviation industry

Alarm fatigue is a form of information overload, which is a well-studied topic in aviation.^19,20^ This problem has been dealt with in the aviation industry through many iterations of system redesign over the years in the pursuit of safety and reliability. At the beginning of the 20th century, the many sensors, clocks and displays in large aircraft required multiple personnel (pilots, a navigator and a flight engineer) to monitor and operate aircraft systems. In contrast, the cockpits of modern aircraft are now sufficiently automated and the information is structured in such a way that the cognitive load can be handled by the pilots alone without a flight engineer or navigator on the aeroplane.^21,22^ In modern aviation, the alarm fatigue problem is carefully managed by engineers and pilots working cooperatively to agree upon exactly what needs to be alerted to the pilot from all aircraft systems and what does not. Agreed alarms are then placed in a hierarchy, with many events being reported only as ‘cautions’ or ‘advisories’ on a screen, but without any auditory alert. This allows pilots to deal with the fundamental needs of flying the plane, rather than being continuously distracted by alarms. Crews are typically able to manage one issue at a time—the one with the currently highest priority—and determine their mitigation strategy. Even an event as apparently serious as an engine failure in a modern commercial multi-engine aircraft does not result in a top-level alarm with an auditory alert, but only a caution. This is in stark contrast to the multitude of trivial alarms that constantly sound in the ICU.^10^

The healthcare literature contains ongoing efforts to improve the quality of clinical alarms and how they are presented. However, the great majority of these efforts remain consistent with the single-device paradigm, as they typically involve signal and algorithm optimisation within individual devices or for specific sets of parameters.^23,24^ While improved specificity and artifact reduction in a number of areas have been achieved with these approaches, they offer little ability to manage or prioritise alarms between devices for all devices attached to a patient.^24–26^ By ignoring the information from other devices and systems, these approaches can only attempt to perfect the nature and presentation of alarms from within the single device, rather than understanding the alarm within the wider clinical context associated with a particular patient. This implies a false dichotomy in alarm processing: an alarm is either wrong (no priority) or right (highest priority), rather than considering the alarm as information with a particular priority relative to many other alarms. Nonetheless, such approaches are important steps to achieve our shared goal of higher-quality alarms and should be considered, but we believe that the total number of auditory alarms in the ICU must be reduced by at least one order of magnitude from current levels, and this is not possible under the single-device paradigm. The networked-device paradigm, by contrast, considers alarm design from a systems perspective, which looks beyond individual devices. The same alarm signals can be issued by individual medical devices, but rather than alarming at the bedside, alarm signals will be passed to the network, which will then manage and prioritise alarms across the entire network, thus having the ability to reduce spurious audible alarms significantly.^9,27,28^ Many medical devices already have Wi-Fi capability and some capacity for sharing information, and industry frameworks for integrating medical devices have been developed.^29,30^ Even without hardware changes, many of these devices are capable of being networked and integrated further, only requiring relatively straightforward software changes. As the networked-device paradigm includes devices from various manufacturers, international communication standards and protocols are necessary to ensure a safe and reliable network.^31^ Hence, we are working with equipment manufacturers to align communication protocols and make use of existing interoperability standards.^32^

## Fundamental needs: Maintaining situational awareness

However, creating virtual networks of devices is not enough—an effective strategy for the management and prioritisation of alarm data is critical within the networked-device paradigm—since if done poorly, a network of devices could lead to just as many if not more alarms than the conventional approach.^33^ In applying a similar design strategy as used in the aviation industry to manage alarms, we have to consider what information is necessary to understand what is happening to our patients so that we can keep them safe and guide them towards recovery. However, aviation is a different industry from healthcare. Pilots have almost full control over a single airplane from within the cockpit, whereas ICU work processes are more fragmented between devices, locations and different team members. Although the designed solution in aviation cannot be directly adopted by the ICU, the underlying design principles can be. In other words, we need to identify the fundamental needs to maintain situational awareness in healthcare providers during patient care. An overload of information will result in a loss of situational awareness. So, we need to prioritise the available information and then restrict it to an amount that can be dealt with by the healthcare provider. All other information should be available only on demand or via notifications on a screen without auditory alerts. For example, seeing the multiple electrocardiograph (ECG) lead traces might be a high priority in a patient with ischaemic heart disease. However, for someone with a respiratory problem, the second ECG lead may be of less importance to maintain situational awareness. Similarly, a septic patient may initially suffer from severe haemodynamic instability, whereas that same patient may be much more stable after several days. We thus need to design a system-wide solution which allows for decisions to be made about what information has what priority in which situation.

## Hide secondary information: Automate monitoring and alarming

Information can be hidden, or reported by silent notifications, if ICU nurses and physicians know that it is safe to do so, creating a healing and quiet patient environment. A computer system may provide such safety when it continuously monitors clinical information, and produces an auditory alarm in a timely way only when patients cross a threshold and deteriorate. The monitored data can be hidden until the threshold is crossed because the healthcare providers can consider it to be *safe by default*. For example, when lung-protective tidal volumes are achieved with acceptable ventilatory pressures, CO_2_ levels and SpO_2_ levels, there may be no need to display this information. However, we need to consider the safety at a system level rather than at the level of individual devices. Current solutions use simple alarm thresholds within individual devices; a parameter that goes out of bounds on each device will trigger an alarm to which a healthcare provider needs to respond. This results in most alarms in an ICU being either false or non-actionable, thus greatly increasing the number of alarms and alarm fatigue.^34,35^ Widening alarm thresholds—or even filtering particular alarms completely—may at first appear to be an obvious approach to reduce the number of false positive alarms. Unfortunately, a system with wider alarm thresholds is likely to be less safe due to the system being less sensitive to changes in the patient’s condition. ICU nurses and physicians may thus feel less comfortable with information not being in direct sight if alarm thresholds are widened without any other safeguards being in place.^35^

**Figure 1.**
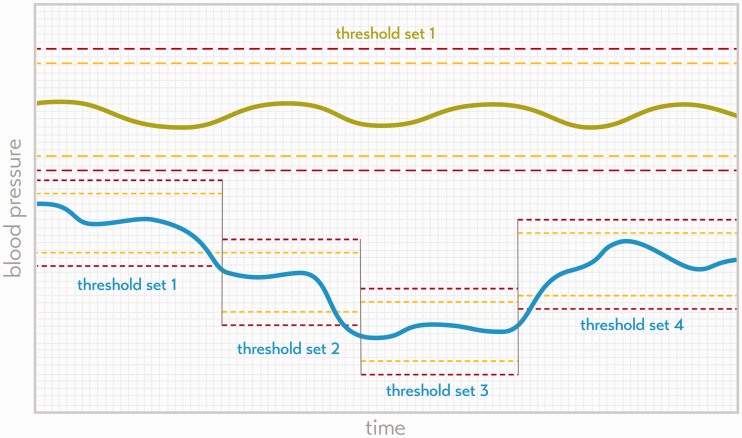
The green line indicates the mean blood pressure of a theoretical healthy patient, who requires only a single threshold set over the full time span (yellow dashed line indicates moderate severity threshold, and the red dashed line the severe alarm threshold for each trace). The blue trace indicates the blood pressure of a theoretical diseased patient with multiple alarm thresholds set over time.

An alternative approach would be to find the optimal set of alarm thresholds for all individual parameters of ICU devices. This may be reasonably straightforward in healthy patients, as the normal range of their physiological parameters will be far from that of a diseased patient, thus allowing differentiation of normal from diseased states. This approach is analogous to the setting of thresholds in aviation; the flight crew starts out with a well maintained or ‘healthy’ aircraft for which clear alarm thresholds can reasonably be inferred. However, the situation in healthcare is much more complex, and a more subtle variation on the aviation alarm-setting approach must be taken. Critically ill patients are continuously in a diseased state which changes in its severity over time. The physiological values that can be considered safe will greatly depend on the diagnosis, the clinical condition of the patient and his or her comorbidities.^26,36,37^ The safety limits of an automated patient monitoring system must therefore be dynamic, not static, and need to adapt or be able to be adjusted to an alternative set of thresholds to suit the clinical condition of the patient (Figure 1) in collaboration with healthcare providers.

## The complementarity of humans and machines

Healthcare providers understand the context of clinical events very well but, like all human beings, have a limited attention span and workload capacity. Alarm systems can monitor many variables closely and continuously and without fatigue but have no understanding of the context of events. The solution is to integrate and prioritise the information from the various devices in order to reduce the total number of alarms and have only the most relevant alarms sound at any given time.

## Comprehensive information

**Figure 2.**
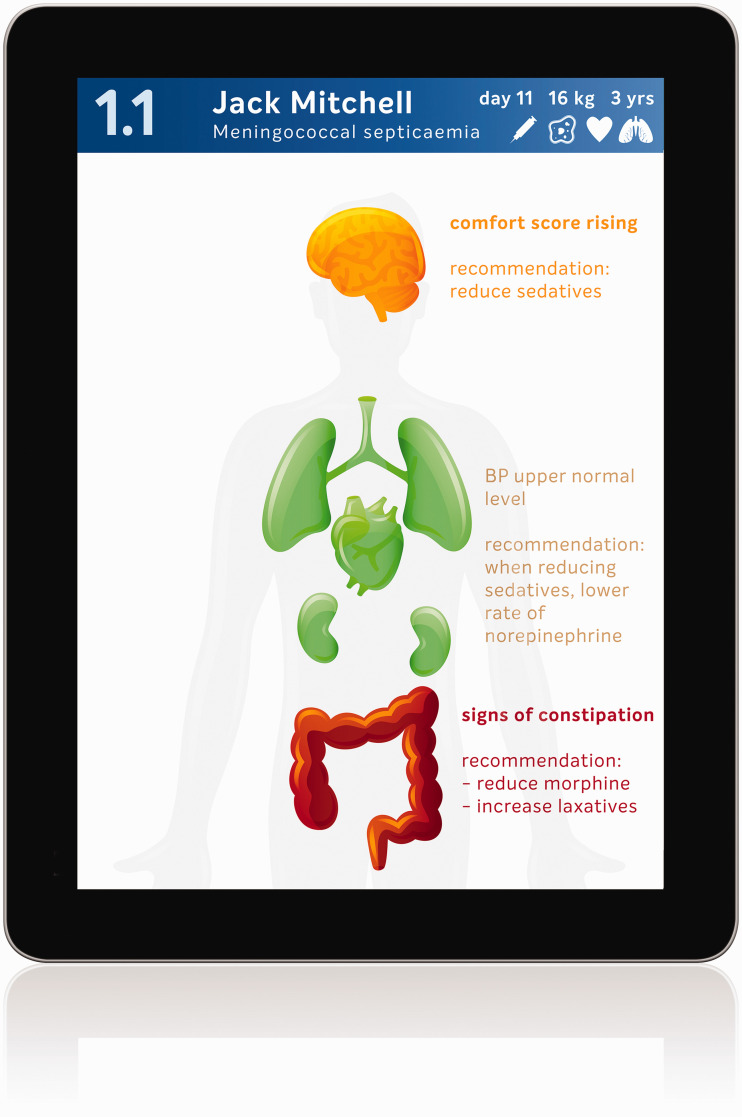
Mock-up of handheld app which could help the healthcare provider to focus attention on the current points at risk using a humaniform display.

If we integrate the information from all devices in the ICU, we could design systems that display the information to healthcare providers in a much more efficient and intuitive way, not only by hiding parameters with a lower priority, but also by combining parameters and integrating these data into more comprehensive and meaningful sets of information. For example, all respiratory and ventilatory parameters—including laboratory values, radiology reports and even breath sounds—could be integrated into a summary status of the clinical condition of the lungs (see an exemplar tablet interface with body overview or humaniform display; Figure 2). In a recent study, physicians and nurse anaesthetists recalled significantly more patient vital signs and reported a reduction in workload when parameters were presented in a humaniform visual representation compared with conventional display methods.^38^

## Comprehensive alarms

The alarms in current ICU devices are generated by each device when their values pass either the moderate threshold (yellow alarm) or the severe threshold (red alarm; Figure 1). The message of the alarm seems simple, for example ‘red alarm: blood pressure low’. However, the message does not necessarily reflect the impact on the patient: a red alarm for low blood pressure may not reflect a similar impact as a red alarm for the detection of asystole on the ECG. Alarm notifications need to be more comprehensive, better prioritised and clinically informed in order to support the situational awareness and decision-making of healthcare professionals. Other work has attempted to suppress alarms and combine data streams but without engaging with clinicians on how this should occur.^39^ Under the networked-device paradigm, new smarter algorithms, operating across individual devices at the system level, are needed to achieve this. These smarter algorithms are currently being created through consultation with clinical experts.^40^ However, in the future, such algorithms could also include approaches using machine learning and artificial intelligence techniques, making use of real patient data in offline big datasets and then applied to live patient devices, but only after appropriate safety testing.^20,25,27^

**Figure 3.**
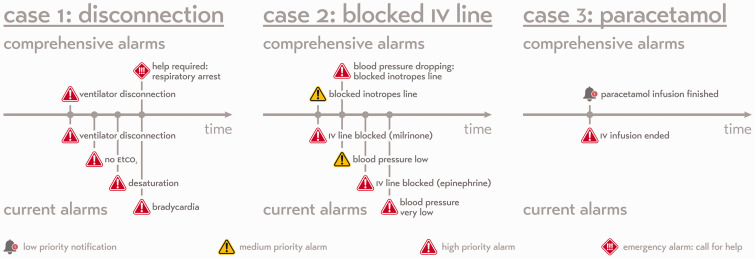
Three theoretical patient case timelines visualising the notifications that are being sent to the healthcare provider in both the current alarm system and in a future comprehensive alarm system. Case 1: An accidental disconnection from the ventilator (a potentially life-threatening event). Within the current system, the patient monitor and ventilator would generate a series of alarms that are not more informative than a single disconnection alarm. A comprehensive alarm system would generate only a single disconnection alarm unless the clinical condition deteriorates. Case 2: A blocked intravenous line due to a distal occlusion. As multiple inotropes are connected to the same line, the inotrope infusion will be reduced. Rather than alarming for each individual drug, the comprehensive system will inform the nurse that the inotropes line is blocked and will escalate that alarm to a higher priority when the patient is affected (a drop in blood pressure). Case 3: In the current system, all infusion pump alarms are communicated with medium to high priority. A comprehensive system would understand that the end of the infusion of paracetamol is not an alarm and should not have priority in demanding attention from the nurse with an auditory alarm.

When all available parameters from all available devices are combined into a system of comprehensive alarms, these can be better prioritised within the work process of the healthcare provider, resulting in substantially fewer but more meaningful alarms (see Figures 2 and 3). Appropriate assignment of priority will result in many alarms being reduced to simple notifications without auditory alerts (theoretical case 3; Figure 3), or to a single auditory alarm instead of many (theoretical case 2; Figure 3). The system will be able to be more informative in its messages and may even be able to suggest an appropriate course of action to the healthcare professional in various circumstances, since integrated patient data yield a more reliable overall picture of the state of the patient’s health (Figure 2). A system that accurately prioritises information across the network of devices attached to the patient is a system that can not only send notifications at appropriate times, but also knows when to withhold unnecessary information and be silent. Such an approach for the settings of the priority for the alarms and alerts of individual patients can also be applied across a network of many patients while still considering the clinical specifics of each. A system of comprehensive alarms could also be able to set alarm thresholds dynamically (Figure 1).^37^ Furthermore, notifications should be sent directly to the correct provider. Sounding an alarm in a patient room when no healthcare professional is present is an unnecessary disturbance for patients and their families—and device standards already exist which allow alarm information to be forwarded elsewhere in this way.^41^

## Safe systems not only safe devices

**Figure 4.**
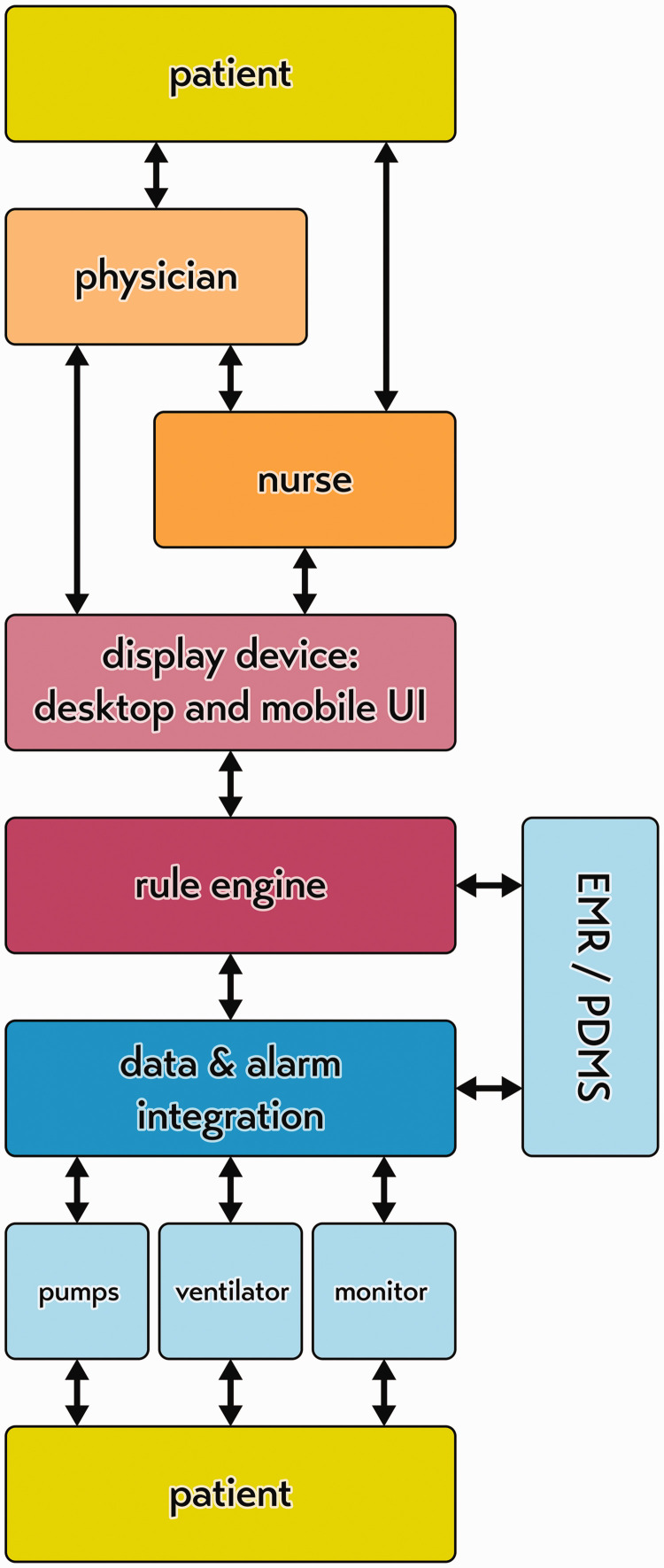
Patient-centred care must be organised around the patient. Safety is critically important and under the networked-device paradigm, the whole chain of devices acts together like an Internet of Things. EMR: electronic medical record; PDMS: patient data management system; UI: user interface.

A systems approach to the design of ICU safety requires that we view the various medical devices as a network of sensors or an Internet of Things, instead of an arbitrary collection of isolated devices.^9,26,33,42,43^ The current legal standards in Europe—the Medical Device Directive and the Medical Device Regulations—remain based on the old paradigm of single-device safety.^11,44^ For example, these standards dictate that an infusion pump alarm should be generated by the device at the bedside of the patient and needs to be at least 45 dBA for one minute.^45^ This technically prohibits a systems approach to patient safety and alarm design, as it views the medical devices as independent alarm generators rather than sensors connected to a network. By contrast, under the networked-device paradigm, alarms should be generated only after data integration from all devices and if the event is warranted as sufficiently high priority, and this will require a chain of devices and steps (Figure 4). This requires the manufacturers of the different medical devices to cooperate and accept that there has to be bidirectional communication between the devices to create distributed alarm systems and the possibility of remote control of devices (consistent with existing initiatives such as Service Oriented Connectivity and Integrating the Healthcare Enterprise).^46,47^ Current regulations lag behind these more progressive connectivity initiatives and so represent a challenge for device manufacturers. However, hospital purchasers who require such safe system features to be in their devices will apply market pressure to manufacturers to make such changes. The alarm features of individual devices could be kept once they have been networked. In the short term, this could be done for regulatory expediency, but in the longer term, this could act as a fail-safe in the unlikely case of network failure, or for the less likely situation where the device is indeed used in isolation. In the longer term, regulatory changes will occur to reflect better the needs of patients and clinicians in modern hospitals and of medical device design. Full compatibility between medical devices is technically possible, even when devices are made by different manufacturers, as is the case in aviation where components of an aircraft cockpit can be made by various manufacturers to exacting specifications in order to maintain compatibility.

## Cooperative redesign

Ultimately, a departure from the single-device paradigm implies that all involved parties—patients, healthcare professionals, device manufacturers, vendors and the regulatory bodies—need to work together to design safe patient alarms at a systems level. This requires cooperation similar to that which occurred between pilots and engineers during the design process that led to the creation of the modern commercial aircraft cockpit. Such a cooperative process in healthcare will ultimately require a shift in the roles and responsibilities of many individual parties.

The second and more major shift of the networked-device paradigm involves changes in the assignment of the responsibility for safety. The current process of safe design and legislation predominately places the technical responsibility with the medical device manufacturers. The responsibility of using such medical devices to deliver safe healthcare lies with the healthcare professionals. However, in order to design safe alarms at a systems level, technical and medical safety become much more intertwined. In our aviation example, the redesign of the cockpit has been accompanied by operational safety initiatives, such as briefings, checklists and crew resource management.^19,48–50^ Also, the medical expert needs to take a greater responsibility for understanding the output of the entire chain of integrated devices. This is only a small extension of what clinicians already do in terms of accounting for the medical context and considering possible artifacts when several medical devices are in use.

## Conclusion

Our current clinical alarm and patient monitoring systems are outdated, and routinely result in information overload for healthcare professionals and unnecessary audiovisual disturbances for patients. A paradigm shift in the way medical device alarms are designed is overdue, and healthcare professionals, device manufacturers, vendors and policymakers need to appreciate that our current patient data infrastructure is still based on a single-device safety paradigm which is now obsolete in modern hospitals. As healthcare professionals, we need to work together with patient organisations, suppliers of medical devices and information technology infrastructure staff. This process has begun at the Academic Medical Centre Utrecht and Erasmus Medical Centre. We believe the future of all clinical alarms, patient monitoring systems and medical devices lies in the networked-device paradigm, comprising comprehensive alarm notification systems and the integration, prioritisation and optimisation of patient information across multiple devices. Such an approach has substantial potential to improve the quality and safety of patient care and the working conditions of healthcare personnel.
